# Machine learning-based risk of pulmonary embolism in stroke patients with lower extremity deep vein thrombosis construction and validation of a prediction model

**DOI:** 10.3389/fneur.2026.1710381

**Published:** 2026-02-26

**Authors:** Li Wu, Luo Yefangxin, Rong Liu, Wei Chen, Wanting Shi, Qiong Qin, Darong Lu, Jiexin Sheng

**Affiliations:** 1 Department of Neurosurgery, Affiliated Hospital of Zunyi Medical University, Zunyi, Guizhou, China; 2 School of Nursing, Zunyi Medical University, Zunyi, Guizhou, China; 3 Department of Nursing, The Second Affiliated Hospital of Zunyi Medical University, Zunyi, Guizhou, China

**Keywords:** early warning model, lower extremity deep vein thrombosis, machine learning, pulmonary emboli, stroke

## Abstract

**Background:**

Up to 42% of stroke patients are susceptible to lower extremity deep vein thrombosis (DVT). The dislodgment of thrombus in deep veins of stroke patients can develop into fatal pulmonary embolism (PE), which has insidious onset and high mortality rate, and the risk factors of PE in stroke DVT are not yet known by clinical staff, which makes it easy to be underdiagnosed and misdiagnosed. In addition, routine CT pulmonary angiography (CTPA) cannot be performed for screening. In this study, machine learning technology was utilized to establish a fast and accurate screening model for pulmonary embolism in patients with lower extremity deep vein thrombosis in stroke.

**Methods:**

In this study, all patients admitted with stroke who developed lower extremity deep vein thrombosis from January 2019 to April 2024 were selected for retrospective study. Patient demographic information, medical history and comorbidities, clinical signs, laboratory indices, hospitalization, and medication were included in the analysis. LASSO regression was utilized for feature dimensionality reduction screening, models were constructed using five machine learning algorithms, and internal validation was completed. Oversampling was performed using the SMOTE algorithm as a way to address the problem of unbalanced sample proportions. Hierarchical k-fold, class weights, random search techniques, and self-stepping policy tuning were used to prevent overfitting and model optimization. Feature attributes were expressed numerically using SHAP.

**Results:**

A total of 337 patients were enrolled in this study, of which, 24 patients developed pulmonary embolism. A total of 11 predictor variables were screened by LASSO regression to construct the model. Among the five machine learning models, the Random Forest Classifier (RFC) model exhibited the best performance, with its area under the curve (AUC) = 0.77, accuracy = 0.721, sensitivity = 0.918, precision = 0.750, and F1 score = 0.826, PR-AUC = 0.895, Brier score = 0.172.all of which were higher than those of the other models. The rankings of the SHAP features, from highest to lowest, were oxygen partial pressure, history of hypertension, D-dimer, serum creatinine, severe lung disease, time in bed ≥72 h, stroke type, heart failure, use of acid-producing drugs, chest pain, and dyspnea.

**Conclusion:**

In this study, five machine learning models were established to assess the likelihood of pulmonary embolism in stroke patients with lower extremity deep vein thrombosis, among which the RFC model performed the best. We can promptly recognize and assess patients at risk of PE based on their SHAP, take early preventive and therapeutic measures, and improve the prognosis of patients.

## 1 Introduction

Global burden of disease data show that stroke is the leading cause of death and disability among adults in China, and the number of current patients is the highest in the world, with five major characteristics: high morbidity, high disability, high mortality, high recurrence rate, and high economic burden (1). In stroke patients, due to hemiparesis, braking, and prolonged bed rest, coupled with the use of medications such as dehydration and hemostasis (2, 3). Upto 42% of stroke patients are prone to induce deep vein thrombosis (DVT) in the lower extremities, which is a more common and serious complication after stroke (4). In stroke patients, thrombus dislodged from the deep vein and reached the pulmonary artery along the blood flow can develop into fatal pulmonary embolism (PE), the incidence of which is as high as 50–60%, accounting for 25–35% of the total morbidity and mortality of stroke patients (5, 6). Currently, the risk factors for the development of PE from DVT in stroke are not known to clinical healthcare professionals; in addition, the onset of PE is insidious, and the rates of missed diagnosis, misdiagnosis, and mortality are extremely high (7)^.^ Clinical CT pulmonary angiography (CTPA) in DVT patients is the preferred method for diagnosing PE, but it is not possible to perform routine CTPA screening because of its expensive cost increasing the economic burden of patients. With the advent of the era of informationization and data science, machine learning algorithms have been widely applied to clinical predictive modeling research at home and abroad. However, there is a lack of machine learning-based risk prediction models for early warning of pulmonary embolism risk in patients with lower extremity deep vein thrombosis in stroke. In this study, several algorithms based on machine learning will be used to establish, evaluate, and compare pulmonary embolism risk prediction models for stroke patients with lower extremity deep vein thrombosis, which will provide a reference for clinical decision-making.

## 2 Methods

### 2.1 General information

This study was approved by the Ethics Committee of the hospital and selected all the patients with stroke who developed lower limb deep vein thrombosis who received by the Affiliated Hospital of Zunyi Medical University from January 2019 to April 2024. Inclusion criteria: ① patients who met the diagnostic criteria of the WTO, the revised diagnostic criteria of the Fourth National Academic Conference on Cerebrovascular Disease, or the diagnostic criteria of the Chinese Guidelines for the Diagnosis and Treatment of Acute Ischemic Stroke 2018 (8); ② patients who were further diagnosed as having a stroke by cranial CT or MRI; and ③ patients who were diagnosed as having a DVT of the lower limbs by venography or Doppler ultrasound, with diagnostic criteria referring to the 2018 American Society of Hematology [American Society of Hematology (ASH)] guidelines for the diagnosis of venous thromboembolism; ④ Age ≥ 18 years; ⑤ Patients and their families were informed about this study and signed an informed consent form. Exclusion criteria: ① patients with lower limb DVT or PE before or within 24 h of admission; ② patients with admission time ≤24 h; ③ those who died within 48 h after admission; ④ patients with incomplete general and clinical data. “Pulmonary embolism (PE) was confirmed by computed tomography pulmonary angiography (CTPA). All patients included in this study underwent systematic screening within 24 h, regardless of symptom status, to minimize clinical bias.”

### 2.2 Methods

Patient demographic information (gender, age, height, weight, BMI, days in hospital, surgical procedure, stroke type), medical history and comorbidities (history of surgery, smoking, alcohol consumption, hypertension, diabetes mellitus, trauma, heart failure, coronary artery disease, hepatic and renal disease, acute infection/pulmonary infection, severe pulmonary disease, respiratory infection/respiratory failure, hypoproteinemia hemoglobinemia, hyponatremia, gastric mucosal lesions/gastrointestinal bleeding), clinical signs (cough, sputum, dyspnea, chest pain, SBP, DBP, impaired consciousness), and laboratory markers [D-dimer, partial pressure of carbon dioxide, partial pressure of oxygen, pH, N-terminal brain natriuretic peptide precursor, platelet count, leukocyte count, hemoglobin, C-reactive protein, plasminogen time, activated partial thromboplastin time, thrombin time, international blood pressure index (IBPI), time, prothrombin time, international normalized ratio, prothrombin time ratio, fibrinogen, blood glucose, triglycerides, low-density lipoprotein cholesterol, total cholesterol, and admission creatinine], hospitalization (head drain, central venous catheter, invasive arterial blood pressure monitoring, artificial airway, endogastric nutrition, blood transfusion, prolonged fever, bed rest ≥72 h, braking or not, pneumatic compression within 48 h of hospital admission and hemiplegic limb training within 48 h of admission), and medication use (dehydrating agents, diuretics, sedative drugs, vasoactive drugs, and acid-producing drugs).

### 2.3 Model building

① Least Absolute Shrinkage and Selection Operator (LASSO) regression was used for feature dimensionality reduction screening. In this study, predictors were standardized prior to LASSO fitting. Feature selection was performed exclusively on the training data, and collinear variable pairs with an absolute correlation coefficient |*ρ*| > 0.9 were pre-removed. ② Five machine learning algorithms were used: Logistic Regression (LR), Gradient Boosting Classifier (GBC), Random Forest Classifier (RFC), Multi-Layer Perceptron Classifier (MLPC), and Support Vector Machine Classifier (SVC). ③ The dataset is divided into training dataset and test dataset in the ratio of 8:2. ④ Oversampling is performed using the SMOTE algorithm as a way to solve the problem of unbalanced sample proportions. SMOTE-NC (*k* = 5) is performed exclusively within the training set, while the validation and test sets maintain their original distribution (PE 6.7%). ⑤ Use hierarchical k-fold, class weights, random search techniques tuning to prevent overfitting and model optimization. ⑥ The training dataset is cross-validated with tenfold to prevent overfitting of specific datasets. ⑦ Numerical expression of feature attributes using SHAP. A positive SHAP value indicates that the corresponding feature leads to a higher risk of pulmonary embolism, while a negative SHAP value indicates that the corresponding feature leads to a lower risk of pulmonary embolism. The magnitude of the SHAP value indicates the contribution of the feature to the prediction performance.

### 2.4 Statistical analysis

For indicators with missing values less than 20%, various models such as Random Forest and Logistic regression were used to predict the filling of missing data. Continuous variables were expressed as mean ± standard deviation or median with interquartile range (IQR) and as categorical variables with numbers and frequencies. Baseline, clinical characteristics were compared between the pulmonary embolism group and the group without pulmonary embolism using the *t*-test or Mann–Whitney *U* test for continuous variables and the chi-square test or Fisher exact test for categorical variables, as appropriate. Area under the curve (AUC), area under the precision recall curve, accuracy, sensitivity, specificity, precision, and F1 score were calculated to assess model performance.

## 3 Results

### 3.1 Comparison of baseline characteristics and correlation

Among 337 patients with lower extremity deep vein thrombosis in stroke, there were 24 patients with pulmonary embolism. From the baseline characteristics of the patients (Table 1), it can be seen that pulmonary embolism was associated with a history of hypertension in stroke, heart failure, severe lung disease, cough, sputum, chest pain, partial pressure of oxygen, bedtime, and the use of vasoactive medications (*p* < 0.05).

**Table 1:** Comparison of baseline characteristics of included patients [*n* (%), *M* (P25, P75)].

Variables	PE (*n* = 24)	Non-PE (*n* = 313)	*t/χ^2^/Z*	*P*
Gender (%)			1.290	0.256
Female	14(58.3)	145(46.3)		
Male	10(41.7)	168(53.7)		
Age (years)	63.5(56.5,73.5)	66(56,73)	−0.311	0.756
Height	159.5(156.25,167.75)	163(156,17)	−0.792	0.428
Weight	65(60,68.75)	63(57,70)	−0.436	0.663
BMI	24.77(23.16,25.38)	24.18(22.49,25.58)	−1.081	0.280
Days of hospitalization	13(10,24)	18(12,26)	−1.435	0.151
Type of surgery (%)			1.665	0.435
No surgery = 0	8(33.33)	130(41.53)		
Interventional = 1	9(37.5)	80(25.56)		
Non-intervention = 2	7(29.17)	103(32.91)		
Type of stroke			5.598	0.111
Cerebral infarction	15(62.5)	113(36.1)		
Cerebral hemorrhage	7(29.2)	135(43.1)		
Subarachnoid hemorrhage	2(8.3)	56(17.9)		
Aneurysm	0(0)	9(2.9)		
History of surgery	5(20.83)	70(22.36)	0.03	0.862
History of smoking	6(25)	87(27.80)	0.087	0.768
History of alcohol consumption	5(20.83)	74(23.64)	0.098	0.754
History of hypertension (%)			−3.146	0.002
No	12(50)	79(25.24)		
Grade 1	4(16.67)	38(12.14)		
Grade 2	4(16.67)	50(15.97)		
Grade 3	4(16.67)	146(46.65)		
History of diabetes	2(8.33)	34(10.86)	0.002	0.965
Trauma	1(4.17)	5(1.60)	–	0.360
Heart failure	4(16.67)	3(0.96)	–	0.001
Coronary heart disease	4(16.67)	27(8.63)	0.897	0.344
Liver and kidney diseases	2(8.33)	53(16.93)	0.659	0.417
Acute infection/lung infection	19(79.17)	238(76.04)	0.120	0.728
Severe lung disease	2(8.33)	97(30.99)	5.515	0.019
Respiratory tract infection/respiratory failure	4(16.67)	65(20.77)	0.047	0.828
Hypoproteinemia	8(33.33)	122(38.98)	0.300	0.584
Hyponatremia	0(0)	16(5.11)	0.406	0.524
Gastric mucosal lesions/gastrointestinal bleeding	5(20.83)	53(16.93)	0.043	0.836
Cough	3(12.5)	8(2.56)	–	0.036
Sputum	3(12.5)	7(2.24)	–	0.027
Dyspnea	1(4.17)	1(0.32)	–	0.138
Chest pain	2(8.33)	1(0.32)	-	0.014
Admission VTE score	6(5,7.75)	7(5,8)	−0.827	0.408
Left lower extremity muscle strength	3(1.25,4.75)	3(1,4)	−0.444	0.657
Right lower extremity muscle strength	2(0,4)	3(0,5)	−1.542	0.123
SBP	145.46 ± 24.89	141.88 ± 23.79	0.682	0.496
DBP	80(73.5,89)	85(75,93.5)	−0.797	0.425
Impaired consciousness			3.493	0.448
Wakefulness	9(37.5)	123(39.30)		
Drowsiness	7(29.17)	74(23.64)		
Blurred consciousness	3(12.5)	17(5.43)		
Drowsiness	2(8.33)	27(8.63)		
Coma	3(12.5)	72(23)		
D-dimer	2.63(1.47,6.12)	2.31(1.36,3.77)	−0.841	0.400
Carbon dioxide partial pressure	32.85(29.23,38.15)	35(31.95,38.6)	−1.447	0.148
Oxygen partial pressure	76.25(66.33,93.05)	98.2(82.95,112.05)	−3.793	0.000
PH	7.44(7.41,7.47)	7.43(7.41,7.45)	−1.811	0.070
N-terminal brain natriuretic peptide precursor	17,061(6501.75,31352.5)	18,095(7,668,38313.5)	0.763	0.445
Platelet count	204(127.25,261.75)	187(144,233)	−0.382	0.703
White blood cell count	9.815(8.51,12.18)	9.84(7.27,12.16)	−0.259	0.796
Hemoglobin	120.08 ± 17.63	118.43 ± 19.67	−0.400	0.689
C-reactive protein	22.11(13.08,50.15)	27.31(10.36,59.83)	−0.257	0.789
Prothrombin time	10.81(10.28,11.78)	10.657(9.89,11.41)	−1.219	0.223
Activated partial thromboplastin time	25.815(23.04,28.88)	25.91(23,29.05)	−0.376	0.707
Prothrombin time	16.6(15.82,18.38)	17.1(15.9,19.45)	−0.491	0.623
International normalized ratio	0.935(0.85,1)	0.91(0.82,1.02)	−0.319	0.750
Prothrombin time ratio	0.925(0.87,1.00)	0.91(0.84,0.97)	−1.248	0.212
Fibrinogen	4.15(3.33,5.00)	3.85(2.66,5.19)	−0.679	0.497
Glucose	7.19(6.32,8.93)	6.84(5.50,8.32)	−1.312	0.190
Triglycerides	1.64(0.91,3.67)	1.54(1.03,2.28)	−0.534	0.594
LDL cholesterol	2.66(2.11,3.03)	2.55(2.14,3.03)	−0.053	0.958
Total cholesterol	4.365(3.15,5.05)	4.4(3.78,4.99)	−0.732	0.464
Admission creatinine	71.5(53,96)	72(58.5,90)	−0.250	0.803
Erythrocyte specific volume	1.1(0.353,3.04)	0.4(0.35,1.1)	−1.647	0.099
Head drains	6(25)	85(27.16)	0.053	0.819
Central venous catheterization	8(33.33)	97(30.99)	0.057	0.811
Invasive arterial blood pressure monitoring	9(37.5)	125(39.94)	0.055	0.814
Artificial airway	12(50)	146(46.65)	0.101	0.751
Gastrointestinal nutrition	12(50)	170(54.31)	0.167	0.683
Blood transfusion	7(29.17)	79(25.24)	0.181	0.671
Prolonged fever	5(20.83)	85(27.16)	0.455	0.500
Bed rest≥72 h	12(50)	243(77.64)	9.246	0.002
With or without braking	4(16.67)	84(26.84)	1.195	0.274
Pneumatic compression within 48 h of admission	10(41.67)	106(33.87)	0.601	0.438
Integrated hemiplegic limb training at 48 h	5(20.83)	57(18.21)	-	0.784
Dehydration	13(54.17)	201(64.22)	0.972	0.324
Diuretics	9(37.50)	78(24.92)	1.842	0.175
Sedative drugs	11(45.83)	118(37.70)	0.624	0.430
Vasoactive drugs	14(58.33)	239(76.36)	3.870	0.049
Acid-forming drugs	14(58.33)	228(72.84)	2.318	0.128

Fisher’s exact probability method; Continuous variables are expressed as the mean ± standard deviation (±s) or median (quartile spacing) *M* (P25, P75), and counting data are expressed as percentages. *P* < 0.05 indicates statistical significance. SBP, systolic blood pressure; BMI, Body Mass Index; VTE, venous thromboembolism; DBP, diastolic blood pressure.

### 3.2 LASSO regression screening predictor variables

**Figure 1:**
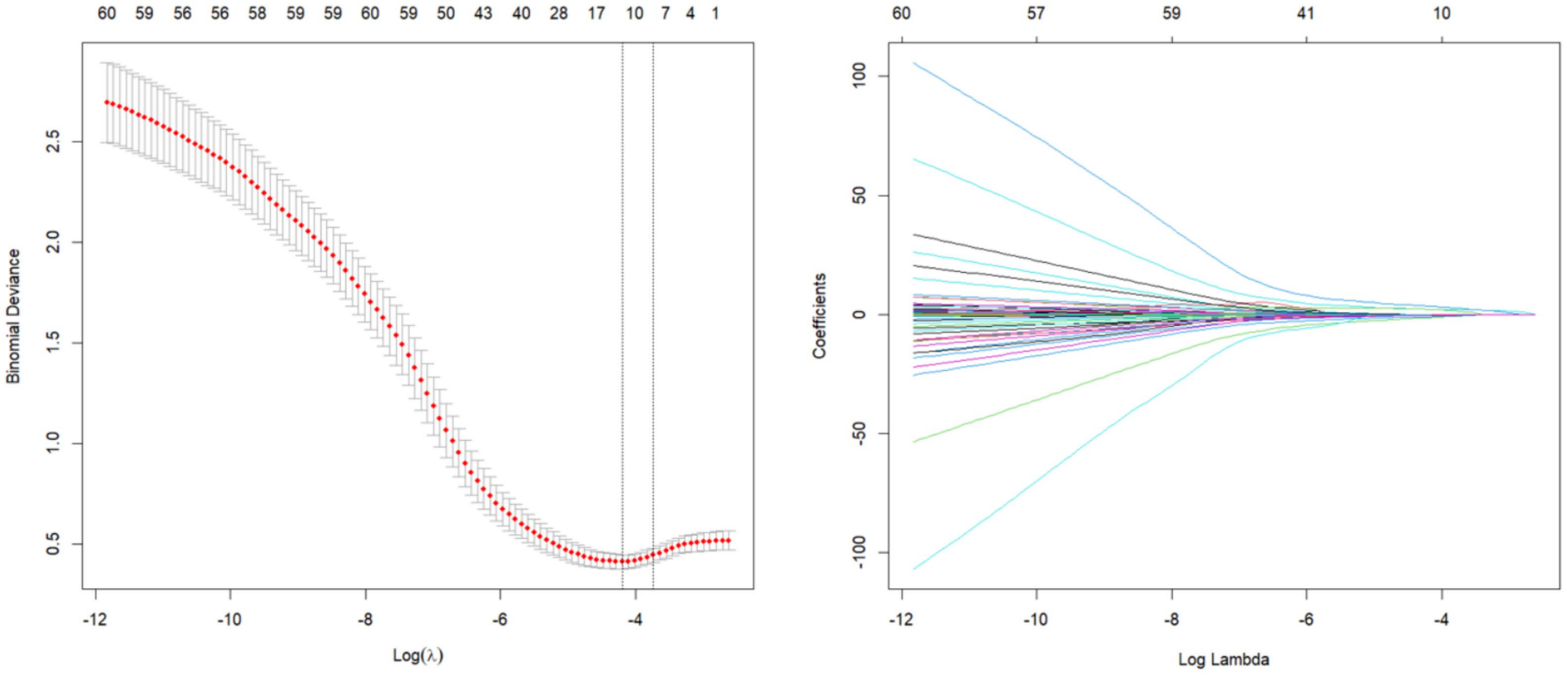
Selection of characterizing variables based on LASSO regression.

**Figure 2:**
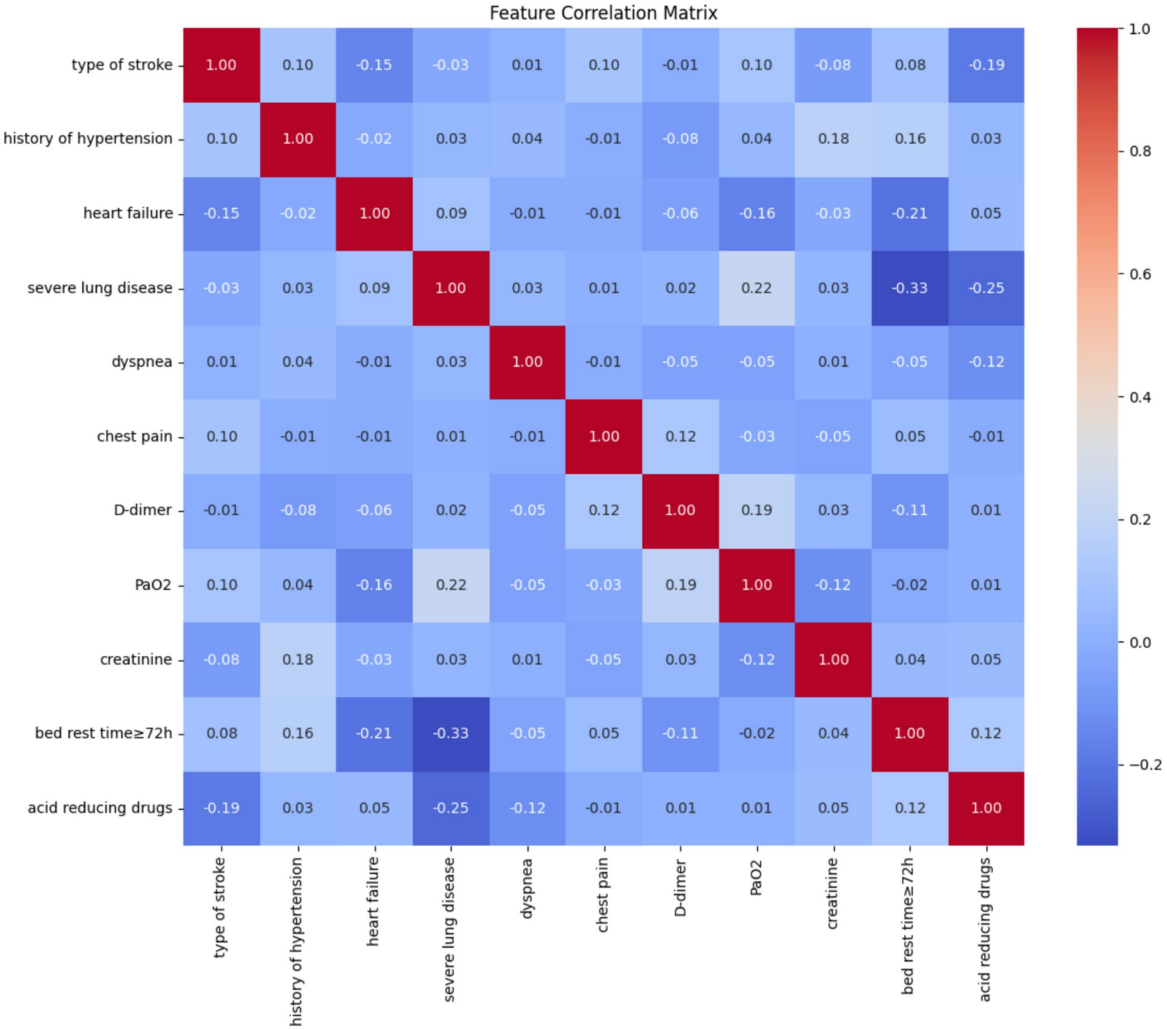
Characteristic correlation rectangles for each variable.

In order to avoid multicollinearity among variables, LASSO regression was used for feature screening, and LASSO regression was performed on the variables, and as the penalty coefficient *λ* increased, the independent variables of the model gradually decreased, and two penalty coefficients, lambda.1se (*λ* = 0.024) and lambda.min (λ = 0.015), were determined by 10-fold cross-validation. In this study, based on the regression coefficients under lambda.min values, a total of 11 predictor variables were finally screened from all variables to construct the model (Figure 1), including stroke type, history of hypertension, heart failure, severe lung disease, dyspnea, chest pain, D-dimer, partial pressure of oxygen, serum creatinine, bed rest ≥72 h, and use of acid-producing medications (regression coefficients for each predictor variable were −0.095, −0.209, 1.947, −0.597, 1.395, 2.587, 0.002775967, −0.009, 0.002904157, −0.503, −0.265660080). The correlation between the variables (Figure 2) showed a strong positive correlation (0.9) between heart failure and stroke type, which means that when one condition occurs, the other is also likely to occur. Secondly, there was also a strong positive correlation between dyspnea and chest pain (0.55), which may represent the fact that these two symptoms often occur together.

### 3.3 Construction and evaluation of machine learning models

**Figure 3:**
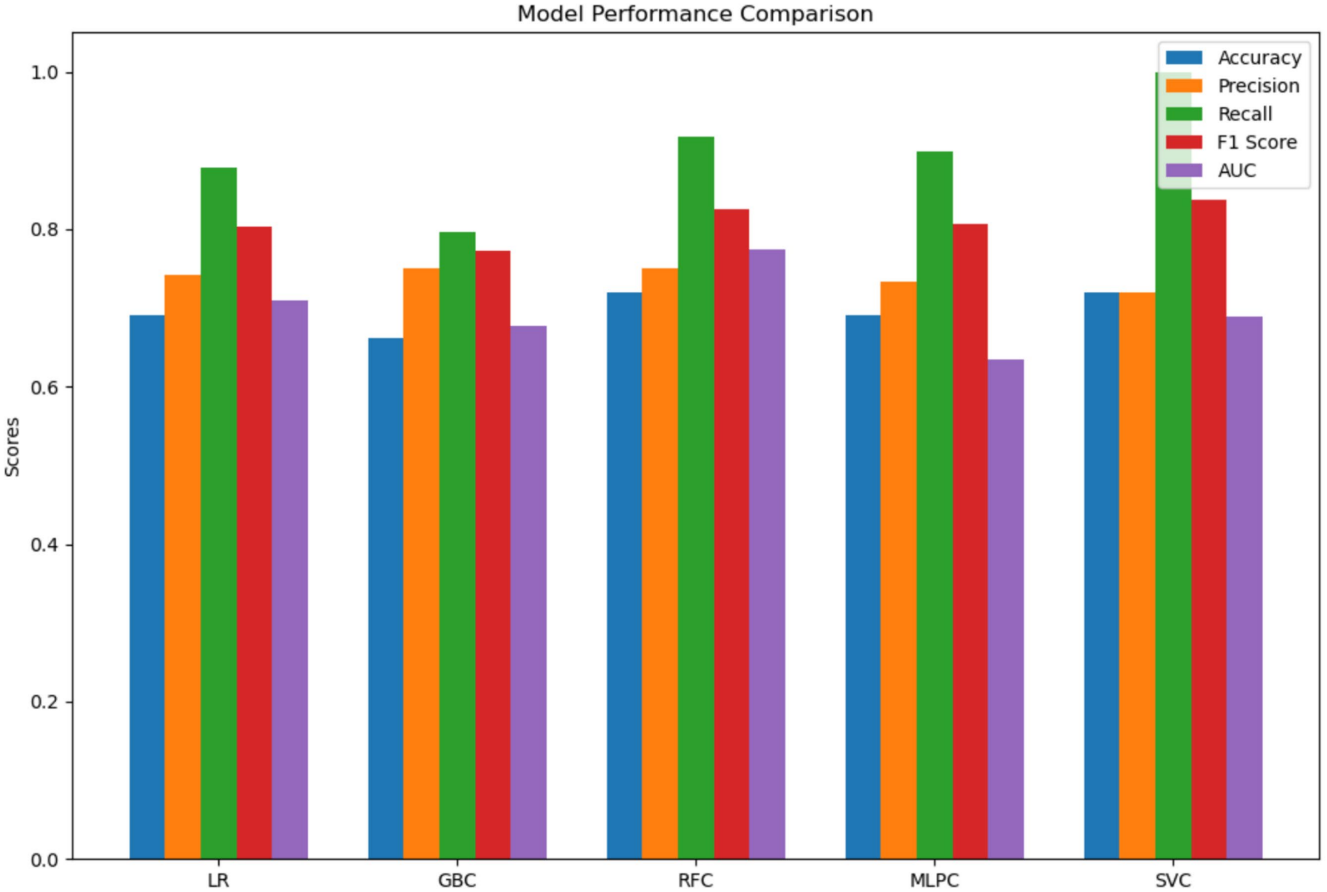
Histogram comparing the performance of the models.

**Figure 4:**
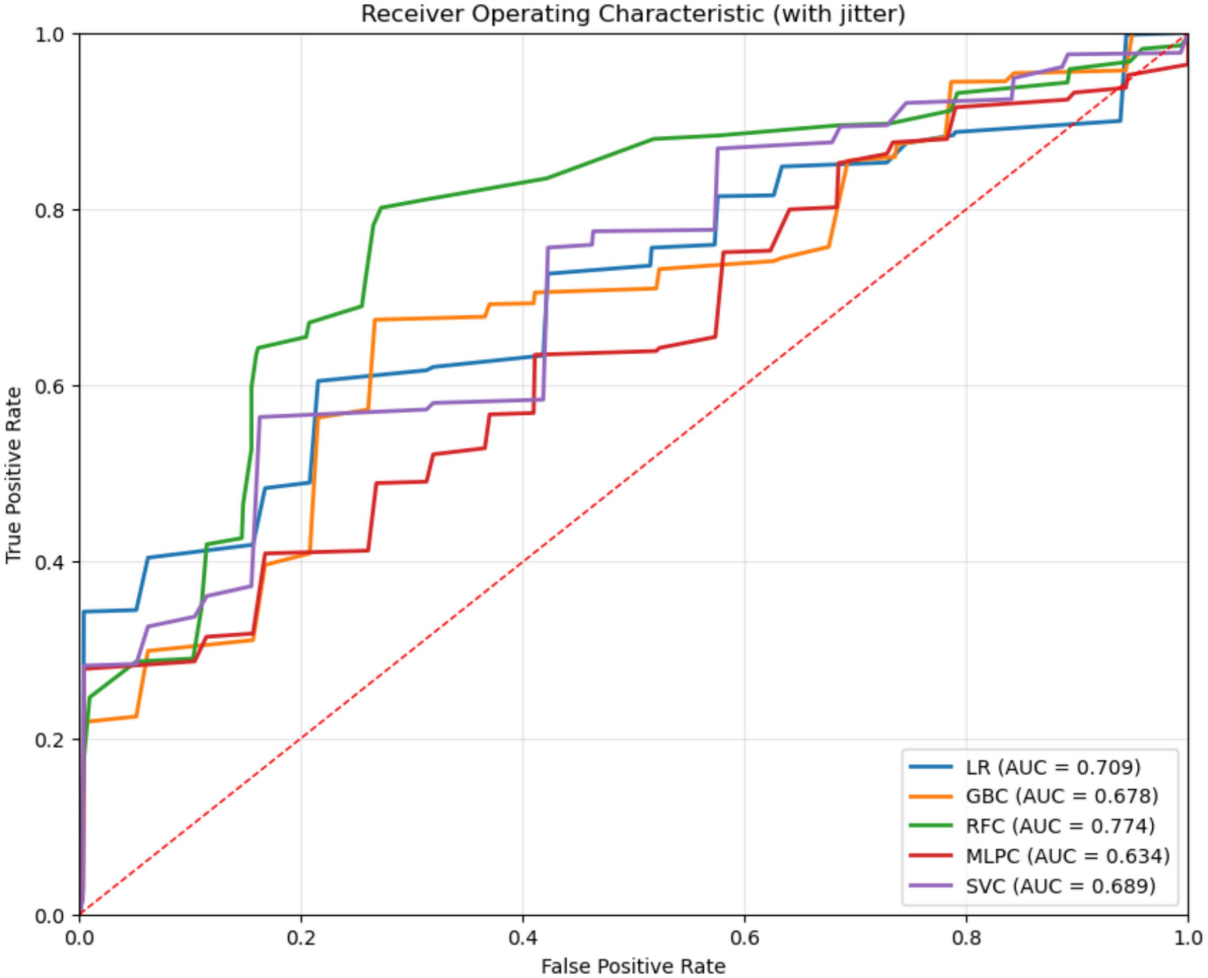
Characterization curves of subjects’ work for five machine learning models.

**Figure 5:**
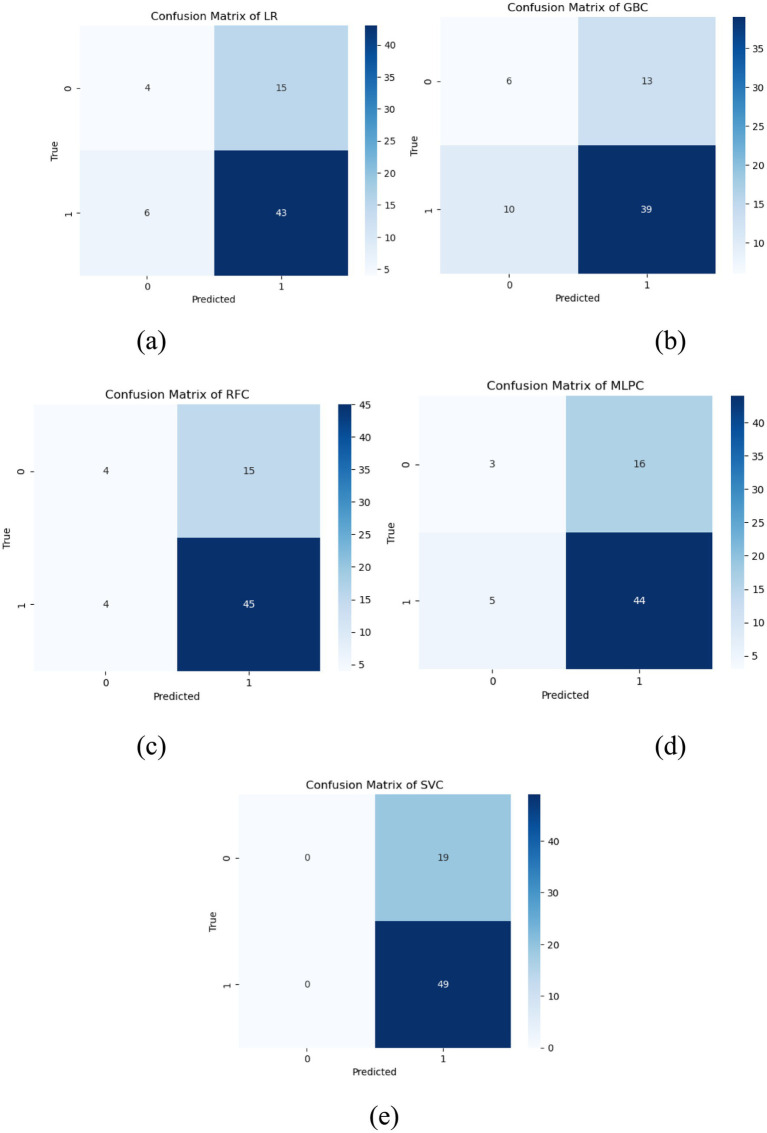
Confusion matrix plots for each model. Five labeled confusion matrix heatmaps **(a-e)** compare classification results of LR **(a)**, GBC **(b)**, RFC **(c)**, MLPC **(d)**, and SVC **(e)** models, each displaying true versus predicted values with counts and a color intensity bar for reference.

**Figure 6:**
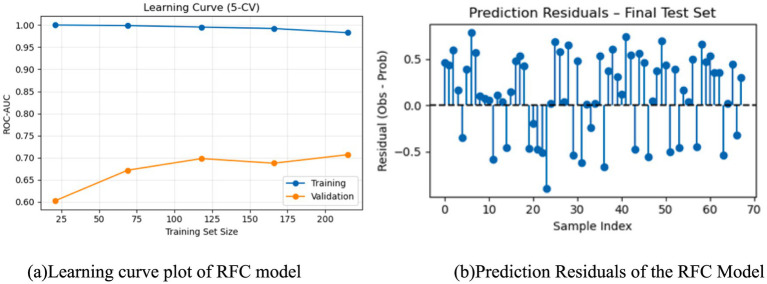
Evaluation of the RFC model. Learning curve plot **(a)** showing ROC AUC for a random forest classifier with training scores near one and validation scores increasing from 0.6 to about 0.7 as training set size grows. Scatter plot of prediction residuals for the final test set **(b)** displays residuals for individual samples clustered around zero with some variation both above and below the axis.

**Figure 7:**
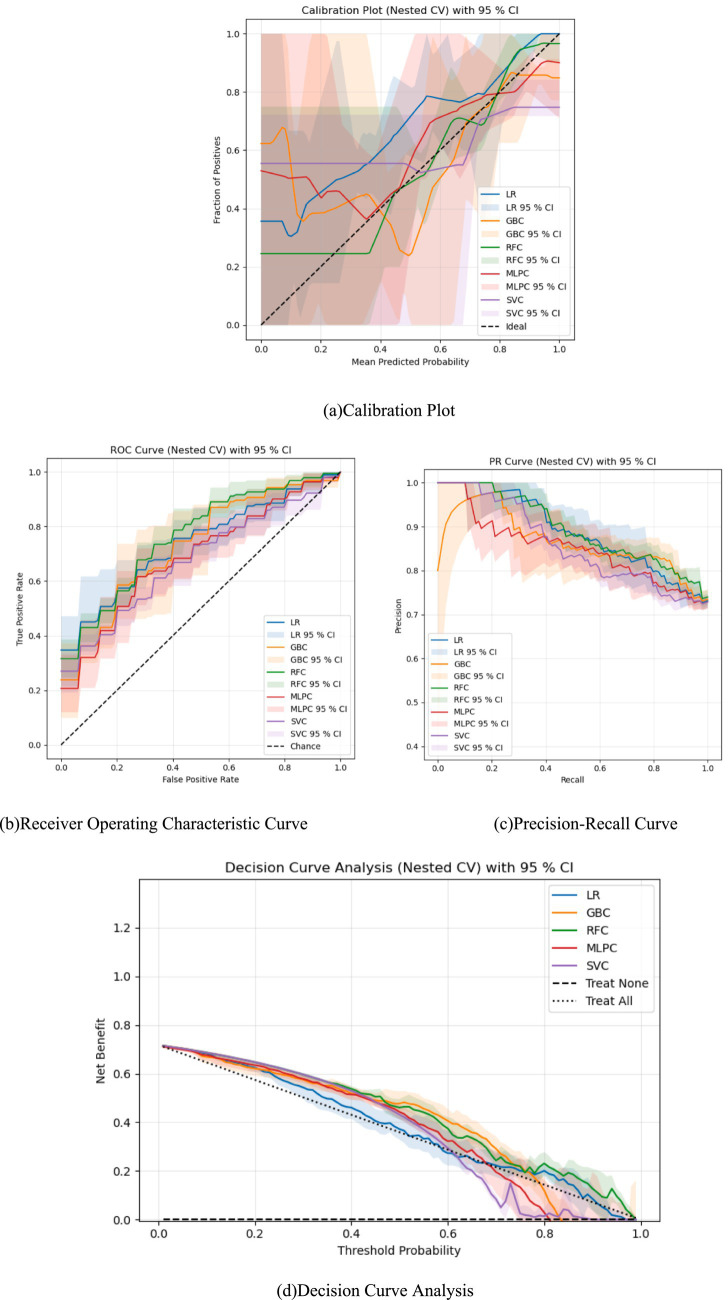
Evaluation of each model. Four-panel figure showing evaluation of machine learning classifiers. Panel **(a)** calibration plot compares predicted versus observed probabilities with shaded ninety-five percent confidence intervals. Panel **(b)** receiver operating characteristic curve depicts true versus false positive rate for each classifier. Panel **(c)** precision-recall curve illustrates precision versus recall performance. Panel **(d)** decision curve analysis shows net benefit across threshold probabilities. Each panel includes results for five classifiers with corresponding model labels and legends.

The 11 features screened by LASSO regression analysis were incorporated into the machine learning model, which was adjusted using 10-fold cross-validation, grid search, parameter tuning, and self-stepping learning strategy to improve the accuracy of the model, and the area under the curve (AUC), accuracy, sensitivity, precision, and F1 scores of each model were shown in Table 2, and the RFC performed the best, with the area under the curve (AUC), accuracy, sensitivity, precision and F1 scores were higher than the other models (Figure 3). The AUC values of each model in descending order were RFC (0.774), LR (0.708), SVC (0.688), GBC (0.6777), and MLPC (0.633) (Figure 4), and the confusion matrices of each model are shown in Figure 5, with RFC having the highest average accuracy. The Evaluation of the RFC Model is shown in Figure 6. The Evaluation of the RFC Model is shown in Figure 7. The learning curve is a tool used in machine learning to evaluate model performance and diagnose model problems. It shows how the performance of the model on the training and validation sets changes as the training samples increase. The learning curve allows us to understand whether the model is overfitting, underfitting or performing well. As the sample size increases, the RFC model training scores and cross-validation scores are higher and the difference between the two is not large, indicating that the model may be overfitting and needs to increase its generalization ability. Taking all the considerations into account, the RFC model was finally selected in this study for visualization and analysis and application of features.

**Table 2:** Comparison of predictive performance metrics for five machine learning algorithms.

Model	AUC	Accuracy	Sensitivity (recall)	Precision	F1 score
LR	0.709	0.691	0.878	0.741	0.709
GBC	0.678	0.662	0.796	0.750	0.678
RFC	0.774	0.721	0.918	0.750	0.774
MLPC	0.634	0.691	0.898	0.733	0.634
SVC	0.689	0.721	1.000	0.720	0.689

Best model is: RFC with AUC: 0.77.

### 3.4 Importance analysis of features

**Figure 8:**
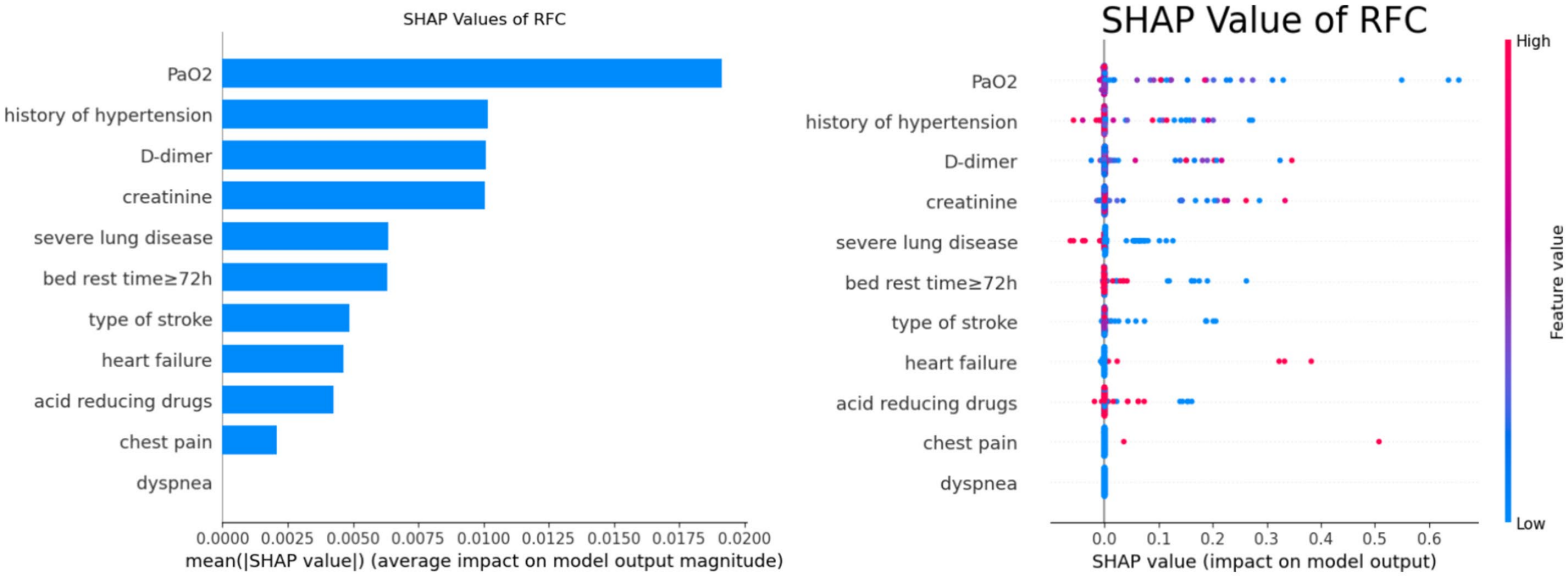
SHAP feature analysis of RFC.

In this study, important features for predicting DVT were identified using the SHAP values of the best predictive model, RFC. Figure 8 shows a summary of the features of SHAP, which were analyzed according to feature importance for risk factors affecting DVT. The features ranked from highest to lowest were oxygen partial pressure, history of hypertension, D-dimer, serum creatinine, severe lung disease, time in bed ≥72 h, stroke type, heart failure, use of acid-producing drugs, chest pain, and dyspnea.

## 4 Discussion

Stroke patients are at a heightened risk for lower extremity deep vein thrombosis (DVT) due to factors such as central nervous system damage, limb dysfunction, prolonged immobility, reduced blood flow, and a hypercoagulable state, which can lead to thrombus dislodgement and pulmonary embolism (9, 10). The diagnostic challenges associated with pulmonary embolism often result in missed or misdiagnosed cases, delaying treatment and worsening prognosis (11, 12). In our study of 337 stroke patients with lower extremity DVT, we identified 24 cases of pulmonary embolism, yielding an incidence rate of 7.12%, consistent with prior research (13, 14). To address sample imbalance, we applied the SMOTE algorithm for oversampling, ensuring model category balance while preventing overfitting. We examined multiple feature variables, ultimately selecting 11 through LASSO regression, and compared five machine learning models. The Random Forest Classifier (RFC) model demonstrated superior performance, yielding better AUC, accuracy, sensitivity, precision, and F1 scores.

Consequently, RFC was selected as our model for early pulmonary embolism screening in stroke patients with lower extremity DVT. Recent advancements in interpretable AI have enhanced understanding of variable importance in model predictions (15). Utilizing SHAP interpretation allowed us to quantify feature contributions and visualize their impact, facilitating the identification of key clinical variables for risk prediction (16). The most important SHAP-ranked features included partial pressure of oxygen (PaO2), history of hypertension, D-dimer, serum creatinine, severe lung disease, time in bed ≥72 h, and others. Partial pressure of arterial oxygen (PaO2) reflects ventilation-perfusion mismatch caused by pulmonary artery occlusion, which is an early blood gas change observed in PE (17). Our findings indicate that lower PaO2 levels correlate with an increased risk of pulmonary embolism in stroke patients with lower extremity DVT. As an indicator of patients’ oxygenation status, decreased PaO2 reflects impaired oxygen exchange due to pulmonary artery obstruction and is a critical manifestation of pulmonary embolism (18, 19). Other studies support the association between low PaO2 and increased pulmonary embolism risk in specific populations (20, 21). Thus, the model’s emphasis on PaO2 aligns with clinical observations and underlying pathophysiological mechanisms.

D-dimer directly reflects fibrinolytic activation and thrombus burden, and is significantly elevated in cases of pulmonary embolism (22, 23). Our findings indicate that elevated D-dimer levels are associated with an increased risk of pulmonary embolism (PE) in stroke patients with lower extremity deep vein thrombosis (DVT). This is consistent with previous studies showing that high D-dimer levels can increase thrombosis incidence (24, 25). Elevated D-dimer indicates active coagulation and fibrinolytic processes, often suggesting the presence of recently formed and degraded thrombi (26). Given that both PE and DVT are venous thromboembolism events, D-dimer testing is crucial for their diagnosis and risk assessment (27). While chronic DVT may present with relatively low D-dimer levels, PE, as an acute event, typically shows significantly higher levels due to extensive fibrinolytic activation (28, 29). This highlights the model’s efficacy in identifying PE risk in patients with lower extremity DVT. Additionally, our findings show that heart failure is linked to a higher risk of PE in these patients. Cardiac insufficiency and venous return obstruction lead to increased thrombus formation in heart failure patients, while also elevating pulmonary circulation pressure, further raising PE risk (30). The model incorporates heart failure as a key feature, reflecting its significance in PE risk. Severe lung diseases, particularly within the past month, also contribute to the risk of PE by causing impaired lung ventilation, hypoxia, and increased blood viscosity, which promotes thrombosis (31). Such conditions make patients more susceptible to worsening lung function and symptoms following PE (32). Lastly, a history of hypertension adversely affects vascular endothelial function, leading to vascular sclerosis and increased coagulation factor activation, thereby raising thrombosis risk (33, 34). Hypertension is a crucial risk factor for stroke, and comorbid cardiovascular diseases further elevate PE risk (35).

Serum creatinine reflects that renal insufficiency increases the risk of coagulation factor retention and endothelial injury, and also affects the clearance of anticoagulant drugs, thus showing a positive correlation with PE risk (36). Elevated serum creatinine levels in stroke patients are often linked to co-infections, immune system damage, and renal impairment. These conditions can lead to vascular endothelial damage, reduced anticoagulant function, and increased procoagulant factors and blood viscosity, raising the risk of pulmonary embolism (PE) (37). Additionally, renal insufficiency can create a hypercoagulable state and hinder the metabolism and clearance of anticoagulants, reducing their effectiveness. Renal dysfunction often indicates poorer overall patient health and comorbidities, further increasing PE likelihood. Thus, monitoring creatinine levels in stroke patients is crucial for timely preventive and therapeutic interventions. Prolonged bed rest, especially for 72 h or more, is an independent risk factor for venous thrombosis, leading to slow blood flow and blood stagnation in the lower limbs, which can result in DVT (38). Dislodged emboli from DVT can lead to PE. Given that stroke patients experience prolonged bed rest due to limb dysfunction, this becomes a critical indicator for assessing PE risk. Regular physical activity is significant for preventing venous thromboembolism (VTE), although optimal exercise parameters remain unresolved (39). Physically inactive adults are encouraged to engage in regular activity to enhance vascular health. The type of stroke may influence treatment strategies and patient outcomes. Different stroke types impact mobility, vascular status, and coagulation, affecting DVT and PE risk. Research indicates that the incidence of venous thrombosis is two to four times higher in patients with acute hemorrhagic stroke compared to those with acute ischemic stroke (40, 41). Our model factors in stroke type, reflecting its relevance to patient risk profiles. Chest pain and dyspnea are typical symptoms of PE, with studies showing chronic dyspnea occurs in nearly half of stroke patients (42). Despite communication challenges, the presence of these symptoms may indicate PE risk, and incorporating them into the model raises awareness. Additionally, the use of acid-producing medications may suggest peptic ulcers or a bleeding risk (43). Such gastrointestinal bleeding is a complication that requires careful attention during anticoagulation therapy, as it may affect drug absorption and metabolism, indirectly influencing PE risk.

### 4.1 Limitations

This study has several limitations. First, it is based on a single-center cohort and thus requires external validation. Second, the sample size of this cohort was small, thus precluding the generalize ability of our model. Third, the study was retrospective, resulting in the inability to test the performance of the model from other populations. In addition, the ML model needs to be improved by other features, including CT images of the legs of the original stroke lower extremity DVT, which may be more conducive to improving the accuracy and scientific validity of our model. Finally, considering the requirements for real-time deployment associated with the online web platform in the later stage, the LASSO method was used to screen variables in advance in the early stage of this study, so as to construct the model under the same scale of several variables and facilitate clinical promotion and application in the later stage. However, LASSO regression has certain limitations in feature screening — for two variables with high collinearity, the algorithm may randomly eliminate one of them. Future studies need to further explore more reasonable variable screening methods for such research.

Several other limitations that must be addressed relate to biases during data collection, including the following three aspects: ① Imaging bias: the diagnosis of pulmonary embolism (PE) relies on computed tomography pulmonary angiography (CTPA). However, some patients with renal insufficiency refuse contrast enhancement, which may lead to missed diagnosis of mild PE (thus underestimating the incidence of PE). ② Time-window bias: Ultrasonic screening for deep vein thrombosis (DVT) is only performed when physicians suspect that the patient may have lower-extremity deep vein thrombosis, which may miss subclinical DVT with insignificant clinical characteristics. ③ The exact time point of anticoagulant initiation was not recorded, so the impact of treatment delay on outcomes cannot be adjusted for. These issues could be addressed in future studies through a multicenter, prospective design incorporating a unified imaging protocol, serial screening, and time-to-event analysis.

## 5 Conclusion

In conclusion, The RFC algorithm can effectively handle high-dimensional data and exhibits a certain degree of adaptability to non-linear relationships. Trained on real case data, the model provides statistically significant feature importance rankings, with SHAP values enhancing interpretability by quantifying each feature’s contribution to predictions. Key non-modifiable predictors include heart failure and severe lung disease, while modifiable factors show limited clinical applicability for PE prevention. Future research should focus on optimizing data collection for critical predictors and exploring additional risk factors like genetic polymorphisms and biomarkers. This approach can aid healthcare professionals in timely identification and intervention for high-risk patients, ultimately improving outcomes for those at risk of PE.

## 6 Summary

### 6.1 What was already known about the topic?

Research has shown that elevated D-dimer levels indicate increased risk of pulmonary embolism (PE) in patients with lower extremity deep vein thrombosis (DVT), reflecting active coagulation processes. Elevated serum creatinine is recognized as a marker of renal dysfunction associated with a hypercoagulable state, raising PE risk in stroke patients. Prolonged bed rest, common in stroke patients, is linked to venous stasis and increased DVT risk, which can lead to PE. Different stroke types affect mobility and coagulation, with hemorrhagic strokes associated with higher thrombosis rates than ischemic strokes. Additionally, chest pain and dyspnea are known symptoms of PE, though communication barriers can complicate their expression in stroke patients. Lastly, the use of acid-producing medications raises concerns about gastrointestinal bleeding risks during anticoagulation therapy. Understanding these factors is crucial for assessing and managing PE risk in stroke patients.

### 6.2 What has this study added to our knowledge?

This study enhances our understanding of the risk factors for pulmonary embolism (PE) in stroke patients with lower extremity deep vein thrombosis (DVT) by integrating machine learning techniques to identify and validate key predictors. It highlights the importance of elevated D-dimer and serum creatinine levels, prolonged bed rest, specific stroke types, and clinical symptoms such as chest pain and dyspnea as critical risk factors for PE. The study also underscores the complex interplay of these factors and their impact on patient outcomes. Additionally, the use of the Random Forest Classifier (RFC) model algorithm provided a reliable model for early detection of PE risk, demonstrating the utility of interpretable AI in clinical settings. By incorporating SHAP values, this research clarified the contributions of various clinical features, offering actionable insights for healthcare providers to enhance monitoring, prevention, and treatment strategies for PE in vulnerable stroke patients. Overall, the study contributes valuable knowledge that can inform clinical practice and promote better patient management in stroke-related thromboembolic events.

## Data Availability

The raw data supporting the conclusions of this article will be made available by the authors, without undue reservation.
